# Changes in the Prescription of Antibiotics and Phytopharmaceuticals in Children Treated for Acute Upper and Lower Respiratory Tract Infections in Pediatric Practices in Germany in 2013, 2018, and 2022

**DOI:** 10.3390/antibiotics12101491

**Published:** 2023-09-28

**Authors:** Karel Kostev, Louisa van den Boom, Christian Tanislav, Louis Jacob

**Affiliations:** 1 Epidemiology, IQVIA, 60549 Frankfurt, Germany; 2 University Clinic, Philipps-Universität Marburg, 35043 Marburg, Germany; 3 Division of Pediatrics/Pediatric Diabetology, DRK Hospital, 57548 Kirchen, Germany; 4 Division of Pediatric Diabetology, Endocrinology, Metabolism and Obesity, Children’s Hospital, University of Bonn, 53127 Bonn, Germany; 5 Department of Geriatrics and Neurology, Diakonie Hospital Jung Stilling, 57074 Siegen, Germany; christian.tanislav@diakonie-sw.de; 6 Research and Development Unit, Parc Sanitari Sant Joan de Déu, CIBERSAM, ISCIII, Dr. Antoni Pujadas, 42, Sant Boi de Llobregat, 08830 Barcelona, Spain; 7 AP-HP, Université Paris Cité, Lariboisière-Fernand Widal Hospital, Department of Physical Medicine and Rehabilitation, 75010 Paris, France

**Keywords:** acute respiratory tract infections, antibiotics, children, Germany, pharmacoepidemiology, phytopharmaceuticals, prescription

## Abstract

*Background*: Little is known about the recent trends in antibiotic and phytopharmaceutical prescribing for acute upper (URIs) and lower respiratory tract infections (LRIs) in children and adolescents. Therefore, this study investigated changes in the prescription of antibiotics and phytopharmaceuticals in children diagnosed with acute URIs and LRIs in pediatric practices in Germany in 2013, 2018, and 2022. *Methods*: The present retrospective study included children aged 2–12 years diagnosed with acute URIs or LRIs in one of 180 pediatric practices in 2013, 2018, and 2022. The URIs included nasopharyngitis, sinusitis, pharyngitis, tonsillitis, laryngitis and tracheitis, and upper respiratory infections of multiple and unspecified sites, while the LRIs corresponded to bronchitis. The primary outcomes were the proportion of children being prescribed antibiotics and the proportion of those being prescribed phytopharmaceuticals. *Results*: A total of 120,894 children were diagnosed with acute URIs or LRIs in 2013 compared to 116,844 in 2018 and 127,821 in 2022. The prevalence of antibiotic prescription decreased for all diagnoses between 2013 and 2022. This decrease was statistically significant for both 2013–2018 and 2018–2022 for nasopharyngitis, pharyngitis, and bronchitis. Meanwhile, there was a significant increase in the use of phytopharmaceuticals for all diagnoses between 2013 and 2018. The prevalence of phytopharmaceutical prescription decreased slightly between 2018 and 2022, but this decrease was generally not statistically significant. *Conclusions*: The prescription of antibiotics has decreased and that of phytopharmaceuticals has increased in children diagnosed with acute URIs and LRIs in Germany over the last decade. More data are needed to corroborate these findings in other settings.

## 1. Introduction

Upper respiratory tract infections (URIs) are infections involving different anatomical regions such as the sinuses, mouth, nose, pharynx, larynx, and large airways [1], while lower respiratory tract infections (LRIs) refer to infections of the smaller airways and lungs [2]. URIs and LRIs are common disorders in children and adolescents [3,4]. Recent evidence from the Global Burden of Diseases (GBD) study suggests that the annual incidence rate of URIs is higher than 250,000 cases per 100,000 individuals in those under the age of 10 [3]. These infections are associated with a substantial burden and disability [3,4]. Finally, URIs and LRIs occurring in children and adolescents lead to school absenteeism [5,6] and parental loss of productivity [6,7]. In this context, the early, personalized management and treatment of pediatric URIs and LRIs should be a public health priority.

Although respiratory infections in the young are frequently caused by viruses [8], the prescription of antibiotic drugs is relatively common in this population. For example, a study including 194,570 children and adolescents younger than 18 years diagnosed with URIs in outpatient practices in South Korea revealed that 58.7% received a prescription for antibiotics [9]. Similar findings were obtained using the data from 89,800 children with respiratory tract infections followed in general practices in Spain, with between 16% and 75% of them being prescribed antibiotics [10]. The high prevalence of antibiotic use for pediatric respiratory tract infections likely results from multiple phenomena, such as healthcare providers trying to meet the expectations of parents [11], being pressured by parents [12], or having limited knowledge about the prescription of antibiotics in this specific context [13]. There is strong evidence showing that the prescription of antibiotic drugs is not associated with shortened duration of respiratory symptoms and does not decrease the risk of complications in children [14]. Furthermore, antibiotic use can have side effects (e.g., diarrhea and candidiasis) [15] and lead to antibiotic resistance [16]. Phytopharmaceuticals, which belong to the field of complementary medicine, are another common treatment for respiratory tract infections. Based on the European Scientific Cooperative on Phytotherapy (ESCOP), phytotherapy corresponds to the science of the medicinal use of plants and herbal products for the prevention and treatment of diseases [17]. Phytopharmaceuticals are used for an extensive range of conditions, such as hypertension [18], atopic dermatitis [19], and depression [20]. In terms of respiratory tract infections, a cross-sectional survey of 250 parents from Turkey revealed that the prevalence of non-drug therapeutic approaches used for their children with symptoms of viral URIs was 47.2%, and that the most common approach was herbal tea (prevalence of 30.5%) [21]. There is some research suggesting that these molecules, which differ from homeopathy, may be effective treatments for respiratory tract infections [22,23,24].

In recent decades, several studies have shown decreasing trends in the prescription of antibiotics for URIs and LRIs in children and adolescents [25,26,27,28,29,30,31,32,33]. Although the results of these bodies of research are critical, they are subject to several limitations that should be acknowledged. First, most of the studies in question analyzed trends prior to 2010 [25,26,27,28,29,30,31], and their findings may not depict recent changes. Second, a substantial proportion of these studies were conducted in the United States of America [25,26,27,30], and it may not be possible to extrapolate their conclusions to other countries and regions of the world. Third, none of this research studied the concomitant trends in the prescription of phytopharmaceuticals for respiratory tract infections in children and adolescents. Taking these data together, more information is needed on the trends in the use of antibiotics and phytopharmaceuticals for young people with URIs in the last decade.

Therefore, the aim of this study was to investigate changes in the prescription of antibiotics and phytopharmaceuticals in children treated for acute URIs and LRIs in pediatric practices in Germany in 2013, 2018, and 2022. The hypothesis was that antibiotic prescription decreased and phytopharmaceutical prescription increased during the study period. In addition, the present research analyzed the correlation between the prescription of antibiotics and the prescription of phytopharmaceuticals per practice. The hypothesis was that there is a negative correlation between antibiotic prescription and phytopharmaceutical prescription per practice.

## 2. Materials and Methods

### 2.1. Database

The present study used data from the Disease Analyzer database (IQVIA). This database has already been described in the literature [34]. To summarize, the Disease Analyzer database contains demographic data, diagnoses, and prescriptions obtained from general and specialized practices in Germany. The data are collected from the computer systems of the practices involved and sent in an anonymous format to IQVIA every month. Diagnoses are coded using the International Classification of Diseases, 10th revision (ICD-10), while prescriptions are coded using the Anatomical Classification of Pharmaceutical Products of the European Pharmaceutical Market Research Association (EphMRA). The data quality is managed by IQVIA and is based on the assessment of different factors (e.g., the completeness of information and the linkage between diagnoses and prescriptions). The inclusion of general and specialized practices in the Disease Analyzer database is based on several criteria, such as physician age, specialty type, community size, and German federal state. Finally, previous research has shown that the database is representative of private practices in the country [34].

### 2.2. Study Population

This retrospective study included children aged 2–12 years who received at least one diagnosis of an acute URI (ICD-10 codes: J00–J06, excluding J02.0, J03.0, and J05) or an acute LRI (ICD-10 code: J20) in one of 180 pediatric practices in Germany in the years 2013, 2018, and 2022. The URIs included acute nasopharyngitis (common cold) (ICD-10 code: J00), acute sinusitis (ICD-10 code: J01), acute pharyngitis (ICD-10 code: J02), acute tonsillitis (ICD-10 code: J03), acute laryngitis and tracheitis (ICD-10 code: J04), and acute upper respiratory infections of multiple and unspecified sites (ICD-10 code: J06). The URIs did not include streptococcal pharyngitis (ICD-10 code: J02.0), streptococcal tonsillitis (ICD-10 code: J03.0), or acute obstructive laryngitis (croup) and epiglottitis (ICD-10 code: J05), as these infections are usually caused by bacteria [35]. The LRIs included acute bronchitis (ICD-10 code: J20). To be included in the study, the participants had to have complete data on their age and sex, with less than 0.5% of patients having missing data for these demographic variables.

### 2.3. Study Outcomes

The primary outcomes of this study were the proportion of children being prescribed antibiotics (EphMRA ATC code: J1) and the proportion of those being prescribed phytopharmaceuticals in Germany in the years 2013, 2018, and 2022. The identification of the prescribed phytopharmaceuticals was based on the names of the drugs. The phytopharmaceuticals included cineole, dried gentian root, elderflower, eucalyptus oil, garden sorrel, gentian root, ivy leaf, *Pelargonium sidoides* root, primrose flower, primrose flower with calyx, primrose root, rectified lemon oil extract, rectified myrtle oil, rectified sweet orange oil, sorrel, thyme, and verbena. These phytopharmaceuticals accounted for more than 90% of all the prescriptions of phytopharmaceuticals in the Disease Analyzer database.

### 2.4. Statistical Analyses

The demographic and clinical characteristics of the children in the years 2013, 2018, and 2022 were described using N (%) for all the variables except for continuous age, which was described using the median (interquartile range). The prevalence of antibiotic and phytopharmaceutical prescriptions was also assessed in 2013, 2018, and 2022 for all diagnoses combined, by URI and LRI diagnosis (i.e., acute nasopharyngitis, acute sinusitis, acute pharyngitis, acute tonsillitis, acute laryngitis and tracheitis, acute upper respiratory infections of multiple and unspecified sites, and acute bronchitis) in the overall sample, and by age group (i.e., 2–5 and 6–12 years). The differences between 2013 and 2018 and between 2018 and 2022 were assessed using Chi-squared tests for the categorical variables and the Wilcoxon rank-sum test for continuous age. Finally, the correlation between antibiotic prescription and phytopharmaceutical prescription per pediatric practice was analyzed using the Pearson correlation coefficient. A correlation analysis was conducted on the sample merging the years 2013, 2018, and 2022. *p*-values lower than 0.050 were considered to be statistically significant. The analyses were performed using SAS version 9.4 (SAS Institute, Cary, NC, USA).

## 3. Results

### 3.1. Demographic and Clinical Characteristics of the Study Sample

A total of 120,894 children were diagnosed with URIs or LRIs in 2013 compared to 116,844 in 2018 and 127,821 in 2022. The demographic and clinical characteristics of the study sample are displayed in Table 1. The median (interquartile range) age was 5.0 (5.0) (*p*-value < 0.001), while the prevalence of boys ranged from 51.8% in 2013 to 52.4% in 2022 (*p*-value = 0.011). The most common URIs were acute upper respiratory infections of multiple and unspecified sites (65.4–71.1%), acute nasopharyngitis (13.2–15.5%), and acute pharyngitis (11.2–15.7%). In terms of LRIs, the prevalence of acute bronchitis was between 15.0% in 2022 and 21.3% in 2013.

### 3.2. Changes in the Prescription of Antibiotics between 2013 and 2018 and between 2018 and 2022

**Figure 1:**
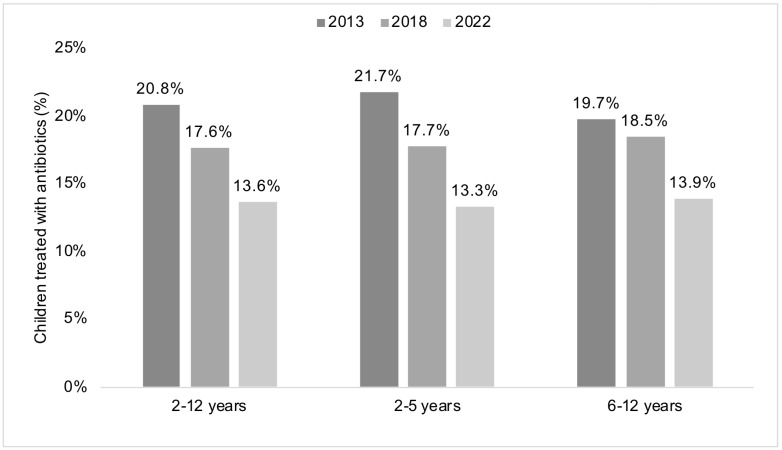
Proportion of children with acute respiratory tract infections who received prescriptions for antibiotics in Germany in the years 2013, 2018, and 2022. Analyses were conducted on the overall sample of children aged 2–12 years (left panel) and in the age groups 2–5 (center panel) and 6–12 years (right panel). In each panel, differences between 2013 and 2018 and between 2018 and 2022 were statistically significant, with *p*-values < 0.001.

The changes in the prescription of antibiotics in children diagnosed with URIs and LRIs in pediatric practices in Germany between 2013 and 2018 and between 2018 and 2022 are shown in Figure 1 and Table 2. When all the diagnoses were analyzed together, antibiotic prescribing significantly decreased in the overall sample between 2013 and 2018 (20.8% versus 17.6%, *p*-value < 0.001) and between 2018 and 2022 (17.6% versus 13.6%, *p*-value < 0.001). Similar findings were obtained in the age groups of 2–5 and 6–12 years. When the diagnoses were analyzed separately, it was observed that the prevalence of the prescription of antibiotics decreased for all diagnoses in the overall sample between 2013 and 2022. This decrease was statistically significant for both periods for acute nasopharyngitis (10.1% in 2013, 8.6% in 2018, and 6.4% in 2022; 2013 versus 2018: *p*-value < 0.001; and 2018 versus 2022: *p*-value < 0.001), acute pharyngitis (21.6% in 2013, 19.7% in 2018, and 18.1% in 2022; 2013 versus 2018: *p*-value < 0.001; and 2018 versus 2022: *p*-value < 0.001), and acute bronchitis (28.9% in 2013, 24.4% in 2018, and 17.5% in 2022; 2013 versus 2018: *p*-value < 0.001; and 2018 versus 2022: *p*-value < 0.001). This decrease in the use of antibiotics was corroborated in the sensitivity analyses conducted on children aged 2–5 years and those aged 6–12 years separately, although the diagnoses for which the decrease was statistically significant for both 2013–2018 and 2018–2022 differed from those mentioned above.

### 3.3. Changes in the Prescription of Phytopharmaceuticals between 2013 and 2018 and between 2018 and 2022

**Figure 2:**
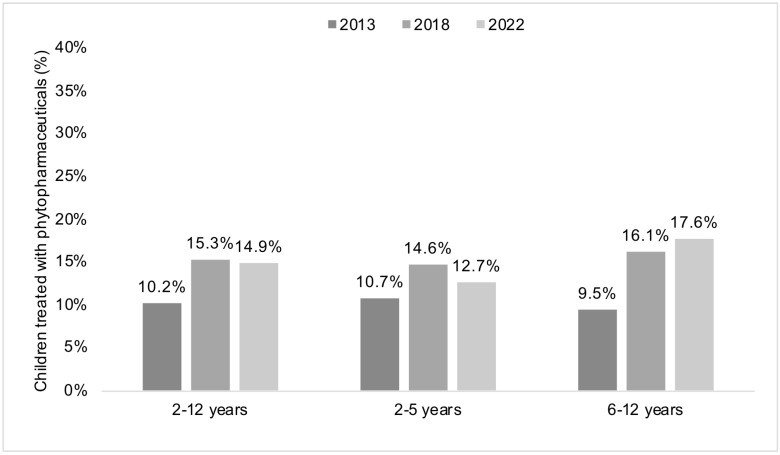
Proportion of children with acute respiratory tract infections who received prescriptions for phytopharmaceuticals in Germany in the years 2013, 2018, and 2022. Analyses were conducted in the overall sample of children aged 2–12 years (left panel) and in the age groups 2–5 (center panel) and 6–12 years (right panel). In each panel, differences between 2013 and 2018 and between 2018 and 2022 were statistically significant, with *p*-values < 0.001.

Figure 2 and Table 3 display the changes in the prescription of phytopharmaceuticals in children diagnosed with URIs and LRIs in pediatric practices in Germany in 2013, 2018, and 2022. In the analyses based on all diagnoses for children aged 2–12 years, there was first an increase in phytopharmaceutical prescribing between 2013 and 2018 (10.2% versus 15.3%, *p*-value < 0.001) and then a decrease between 2018 and 2022 (15.3% versus 14.9%, *p*-value < 0.001). A comparable trend was identified in children aged 2–5 years, but not in those aged 6–12 years, for whom phytopharmaceutical prescribing increased from 9.5% in 2013 to 16.1% in 2018 and 17.6% in 2022. In the analyses investigating the diagnoses separately, there was a significant increase in the use of phytopharmaceuticals in children aged 2–12 years for all diagnoses between 2013 and 2018. The relative increase was more pronounced for acute pharyngitis (4.8% in 2013 and 7.8% in 2018; *p*-value < 0.001), acute bronchitis (8.5% in 2013 and 12.9% in 2018; *p*-value < 0.001), and acute nasopharyngitis (14.2% in 2013 and 21.4% in 2018; *p*-value < 0.001). The prevalence of phytopharmaceutical prescriptions decreased between 2018 and 2022 for all diagnoses, but this decrease was not always statistically significant, and the effect size of this decrease was not as great as that of the increase observed between 2013 and 2018. Similar findings were obtained for children aged 2–5 and 6–12 years, respectively, although a significant increase was observed in the age group of 6–12 years between 2018 and 2022 for acute pharyngitis (8.1% in 2018 and 9.1% in 2022) and acute upper respiratory infections of multiple and unspecified sites (18.4% in 2018 and 19.9% in 2022).

### 3.4. Correlation between Antibiotic Prescription and Phytopharmaceutical Prescription per Pediatric Practice

Table 4 shows the results of the analysis investigating the correlation between the prescription of antibiotics and the prescription of phytopharmaceuticals per pediatric practice in the years 2013, 2018, and 2022, with the data of the three years being merged. While there was no statistically significant correlation for the majority of diagnoses, we did identify a negative and significant correlation between the prescription of antibiotics and the prescription of phytopharmaceuticals for acute sinusitis (Pearson correlation coefficient = −0.27; *p*-value < 0.001) and acute bronchitis (Pearson correlation coefficient = −0.12; *p*-value = 0.013).

## 4. Discussion

### 4.1. Main Findings

This retrospective study, including more than 116,000 children aged 2–12 years with at least one medical consultation at one of 180 pediatric practices in Germany in the years of 2013, 2018, and 2022, showed that the prescription of antibiotics for URIs and LRIs has decreased over time. Meanwhile, the prescription of phytopharmaceuticals increased between 2013 and 2022. Finally, there was no statistically significant correlation between antibiotic and phytopharmaceutical prescriptions per pediatric practice for most respiratory tract infections. To the best of the authors’ knowledge, this is the first study investigating the trends in the prescription of antibiotics in children and adolescents diagnosed with URIs and LRIs in Germany, and it is also the first body of research analyzing the global trends in the prescription of phytopharmaceuticals in this population.

### 4.2. Interpretation of the Findings

The first critical finding of this study was the decreasing trend in antibiotic prescriptions in children diagnosed with URIs and LRIs. This result is in line with previous data obtained in other countries [25,26,27,28,29,30,31,32,33]. For example, a nationwide population-based study of almost 1.7 million pediatric outpatient visits for respiratory tract infections in Taiwan found that the annual prevalence of antibiotic prescriptions decreased from around 18% in 2000 to 4% in 2009 [31]. In another body of research, including 156,187 children and adolescents diagnosed with uncomplicated URIs in Finland, it was observed that the proportion of those being prescribed antibiotics dropped from 18.0% in 2014 to 8.8% in 2020 [32]. In terms of LRIs, similar findings were obtained in a sample of 89,359 individuals under the age of 18 years from the same country, where the prevalence of antibiotic prescription was 37.0% in 2014 and 20.1% in 2020 [33]. This decreasing trend may be explained by the increasing awareness among the medical community, and more particularly pediatricians, regarding the lack of effectiveness of antibiotics for most respiratory tract infections [14], and the potential side effects [15] and antibiotic resistance [16] associated with their use. Although no longitudinal data are available on the attitudes of pediatric trainees towards antibiotic prescription, some preliminary findings are available regarding the attitudes of general practitioner trainees. A study of 2839 general practitioner trainees in Australia revealed that the prescription of antibiotics decreased from 24% in 2010 to 12% in 2019 for URIs and from 84% to 72% for bronchitis/bronchiolitis [36]. These results suggest that the education of health providers regarding the use of antibiotics for respiratory tract infections has evolved in recent years. The decreasing trend in antibiotic prescribing in recent years may also be related to the coronavirus disease 2019 (COVID-19) pandemic. As a matter of fact, a claims-based study of children and adolescents in Germany (N = 9,688,483 participants in 2021) found that the decrease in outpatient antibiotic prescriptions was more pronounced for some months in 2020 compared to prior years, although this effect was temporary [37]. This decrease following the outbreak of the pandemic may have been related to lower access to medical care and partial drug shortages in community pharmacies.

Another major finding was that the prescription of phytopharmaceuticals increased in pediatric practices in Germany between 2013 and 2018 and between 2018 and 2022. To the best of the authors’ knowledge, this is the first study investigating the trends in the use of phytopharmaceuticals for respiratory tract infections in children and adolescents. Before going further, it should be considered that phytopharmaceuticals differ from homeopathic drugs, although both phytotherapy and homeopathy belong to the field of complementary medicine [38]. The increasing trend in phytopharmaceutical prescription may be explained by the fact that some scientific research has suggested that these drugs are effective treatments for respiratory tract infections [22,23,24]. For example, it was observed in a systematic review and meta-analysis, including 11 randomized controlled trials and a total of 2181 children and adolescents with respiratory tract infections, that *Pelargonium sidoides* was an effective and safe molecule [23]. In adults, a study of 14,068 patients diagnosed with acute URIs and LRIs in Germany also found that the prescription of ivy leaf dry extract EA 575 was significantly associated with decreased odds of antibiotic prescription [39]. At the same time, there was a negative association between the prescription of ivy leaf dry extract EA 575, sick leave duration, and subsequent respiratory tract infection diagnoses compared to the prescription of antibiotics. Interestingly, another body of research conducted in the same country, including 234,364 participants with acute respiratory tract infections, also showed that the use of phytopharmaceuticals led to decreased antibiotic prescriptions and decreased odds of prolonged periods of sick leave [40]. Finally, contrary to the initial study hypothesis, we found no statistically significant correlation between antibiotic and phytopharmaceutical prescriptions for most respiratory tract infections per pediatric practice. Interestingly, these two prescribing behaviors were negatively correlated for acute sinusitis and bronchitis, suggesting that health providers prescribing phytopharmaceuticals may be less likely to prescribe antibiotics for these disorders. Nevertheless, further research is warranted to corroborate or invalidate this finding before any firm conclusion can be drawn.

### 4.3. Public Health Implications and Directions for Future Research

The decreasing trend in the prescription of antibiotics for URIs and LRIs observed in pediatric practices in Germany during the last decade suggests that pediatrician awareness about the lack of efficacy and potential deleterious effects of antibiotics has recently increased. However, the use of antibiotics remained high for acute tonsilitis (i.e., 66.7%) and sinusitis (i.e., 27.1%) in 2022, highlighting the fact that public health measures are still needed to promote judicious antibiotic prescription. These public health measures may include, for example, clinical practice guidelines, peer education, and feedback based on local, national, and international indicators [41]. Furthermore, as previous studies have found a negative association between the use of phytopharmaceuticals and the use of antibiotics [39,40], the increasing trend in phytopharmaceutical prescription may further accentuate the decreasing trend in antibiotic prescription. In terms of future research, more studies are needed on the trends in antibiotic and phytopharmaceutical prescriptions in children and adolescents diagnosed with URIs and LRIs in other countries and settings. Furthermore, although the present scientific literature on the efficacy and tolerance of phytopharmaceuticals is reassuring, more real-world data are urgently needed on these molecules.

### 4.4. Strengths and Limitations

The major strengths of this study are its large sample size, the use of real-world data, and the use of data collected over 10 years. Nonetheless, there are also several limitations that should be acknowledged. First, the URI and LRI diagnoses relied on the ICD-10 codes only, and the availability of more information on the severity of these infections might have allowed for more detailed analyses. Second, no data were available on over-the-counter drugs, and different trends may have been observed for these molecules. Third, the children were followed in pediatric practices, and no information was available on those treated in hospitals. Therefore, the findings of the study cannot be extrapolated to hospital settings. Fourth, although the prescription of antibiotics may have been motivated by biological data (e.g., the C-reactive protein concentration in the blood and the presence of bacteria on nasopharyngeal swabs), these data were unavailable and could not be included in the analyses.

### 4.5. Conclusions

In this study conducted in pediatric practices in Germany in 2013, 2018, and 2022, the prevalence of antibiotic and phytopharmaceutical prescriptions in children diagnosed with URIs and LRIs decreased and increased over time, respectively. The decreasing trend in the prescription of antibiotics is reassuring, and efforts should be made towards promoting this trend in the country in the coming years. Although the scientific literature on phytopharmaceuticals is reassuring, further data are warranted on the efficacy and long-term safety of these drugs. Finally, more studies are required in order to corroborate or invalidate these results in other countries.

## Figures and Tables

**Table 1:** Demographic and clinical characteristics of children diagnosed with acute respiratory tract infections in Germany in the years 2013, 2018, and 2022.

Variable	2013	2018	2022	*p*-Value ^a^
N	120,894	116,844	127,821	Not applicable
*Age (in years)*				
Median (interquartile range)	5.0 (5.0)	5.0 (5.0)	5.0 (5.0)	<0.001
2–5	64,283 (53.2)	63,902 (54.7)	66,584 (52.1)	<0.001
6–12	56,611 (46.8)	52,942 (45.3)	61,237 (47.9)	
*Sex*				
Female	58,248 (48.2)	55,988 (47.9)	60,817 (47.6)	0.011
Male	62,646 (51.8)	60,856 (52.1)	67,004 (52.4)	
*Diagnosis*				
Acute nasopharyngitis (common cold) (ICD-10 code: J00)	15,955 (13.2)	18,105 (15.5)	18,774 (14.7)	<0.001
Acute sinusitis (ICD-10 code: J01)	1680 (1.4)	1202 (1.0)	772 (0.6)	<0.001
Acute pharyngitis (ICD-10 code: J02) ^b^	18,959 (15.7)	16,845 (14.4)	14,309 (11.2)	<0.001
Acute tonsillitis (ICD-10 code: J03) ^c^	19,134 (15.8)	15,016 (12.9)	12,716 (9.9)	<0.001
Acute laryngitis and tracheitis (ICD-10 code: J04)	10,539 (8.7)	9414 (8.1)	10,491 (8.2)	<0.001
Acute upper respiratory infections of multiple and unspecified sites (ICD-10 code: J06)	79,099 (65.4)	78,939 (67.6)	90,845 (71.1)	<0.001
Acute bronchitis (ICD-10 code: J20)	25,761 (21.3)	19,729 (16.9)	19,224 (15.0)	<0.001

Data are N (%) unless otherwise specified. Abbreviation: ICD-10 International Classification of Diseases, 10th revision. ^a^ *p*-values were obtained using Chi-squared tests for all variables except for continuous age, for which the Wilcoxon rank-sum test was used. ^b^ Acute pharyngitis did not include streptococcal pharyngitis (ICD-10 code: J02.0). ^c^ Acute tonsillitis did not include streptococcal tonsillitis (ICD-10 code: J03.0).

**Table 2:** Proportion of children with acute respiratory tract infections who received prescriptions for antibiotics by type of diagnosis in Germany in the years 2013, 2018, and 2022.

Diagnosis	2013	2018	2022	*p*-Value for the Difference between 2013 and 2018 ^a^	*p*-Value for the Difference between 2018 and 2022 ^a^
*In children aged 2* *–12 years*					
Acute nasopharyngitis	10.1	8.6	6.4	<0.001	<0.001
Acute sinusitis	32.9	29.1	27.1	0.030	0.325
Acute pharyngitis	21.6	19.7	18.1	<0.001	<0.001
Acute tonsillitis	72.1	72.2	66.7	0.936	<0.001
Acute laryngitis and tracheitis	9.3	8.0	8.2	0.002	0.723
Acute upper respiratory infections of multiple and unspecified sites	9.0	8.1	6.6	0.508	<0.001
Acute bronchitis	28.9	24.4	17.5	<0.001	<0.001
*In children aged 2* *–5 years*					
Acute nasopharyngitis	11.0	8.0	5.8	<0.001	<0.001
Acute sinusitis	26.8	25.6	19.4	0.714	0.112
Acute pharyngitis	24.0	20.7	19.8	<0.001	0.163
Acute tonsillitis	73.5	72.4	67.3	0.108	<0.001
Acute laryngitis and tracheitis	8.9	7.2	7.7	<0.001	0.287
Acute upper respiratory infections of multiple and unspecified sites	9.8	8.7	5.9	<0.001	<0.001
Acute bronchitis	29.9	23.4	16.2	<0.001	<0.001
*In children aged 6–12 years*					
Acute nasopharyngitis	8.9	9.4	7.1	0.261	<0.001
Acute sinusitis	35.2	30.2	30.0	0.015	0.927
Acute pharyngitis	19.5	18.8	16.7	0.196	<0.001
Acute tonsillitis	70.6	71.9	65.9	0.078	<0.001
Acute laryngitis and tracheitis	9.8	9.2	8.7	0.329	0.491
Acute upper respiratory infections of multiple and unspecified sites	8.0	9.5	7.3	<0.001	<0.001
Acute bronchitis	27.1	26.4	19.7	0.280	<0.001

Data are %. ^a^ *p*-values were obtained using Chi-squared tests for all variables.

**Table 3:** Proportion of children with acute respiratory tract infections who received prescriptions for phytopharmaceuticals by type of diagnosis in Germany in the years 2013, 2018, and 2022.

Diagnosis	2013	2018	2022	*p*-Value for the Difference between 2013 and 2018 ^a^	*p*-Value for the Difference between 2018 and 2022 ^a^
*In children aged 2–12 years*					
Acute nasopharyngitis	14.2	21.4	19.0	<0.001	<0.001
Acute sinusitis	45.8	57.0	55.1	<0.001	0.397
Acute pharyngitis	4.8	7.8	7.7	<0.001	0.721
Acute tonsillitis	2.8	4.0	3.9	<0.001	0.510
Acute laryngitis and tracheitis	8.2	12.3	11.2	<0.001	0.010
Acute upper respiratory infections of multiple and unspecified sites	12.5	17.9	17.3	<0.001	0.003
Acute bronchitis	8.5	12.9	12.7	<0.001	0.472
*In children aged 2–5 years*					
Acute nasopharyngitis	15.3	21.0	15.9	<0.001	<0.001
Acute sinusitis	46.4	54.4	52.1	0.038	0.623
Acute pharyngitis	5.3	7.5	6.2	<0.001	0.002
Acute tonsillitis	2.7	3.7	3.5	<0.001	0.370
Acute laryngitis and tracheitis	9.4	12.5	10.0	<0.001	<0.001
Acute upper respiratory infections of multiple and unspecified sites	13.6	17.5	15.0	<0.001	<0.001
Acute bronchitis	8.2	11.3	10.4	<0.001	0.020
*In children aged 6–12 years*					
Acute nasopharyngitis	12.7	22.0	22.6	<0.001	0.404
Acute sinusitis	45.6	57.8	56.2	<0.001	0.543
Acute pharyngitis	4.3	8.1	9.1	<0.001	0.034
Acute tonsillitis	2.8	4.4	4.4	<0.001	0.974
Acute laryngitis and tracheitis	6.6	12.1	12.6	<0.001	0.481
Acute upper respiratory infections of multiple and unspecified sites	11.2	18.4	19.9	<0.001	<0.001
Acute bronchitis	9.2	15.9	16.7	<0.001	0.173

Data are %. ^a^ *p*-values were obtained using Chi-squared tests for all variables.

**Table 4:** Correlation between antibiotic prescription and phytopharmaceutical prescription per pediatric practice in Germany.

Diagnosis	Pearson Correlation Coefficient	*p*-Value
Acute nasopharyngitis	−0.04	0.886
Acute sinusitis	−0.27	<0.001
Acute pharyngitis	0.05	0.310
Acute tonsillitis	0.03	0.587
Acute laryngitis and tracheitis	−0.07	0.187
Acute upper respiratory infections of multiple and unspecified sites	0.03	0.477
Acute bronchitis	−0.12	0.013

This analysis was conducted on the sample merging the years 2013, 2018, and 2022.

## Data Availability

The data used in the present study are available from the corresponding author upon reasonable request.
